# Profiling of Potential Antibacterial Compounds of Lactic Acid Bacteria against Extremely Drug Resistant (XDR) *Acinetobacter baumannii*

**DOI:** 10.3390/molecules26061727

**Published:** 2021-03-19

**Authors:** Phui-Chyng Yap, Noorfazlin Ayuhan, Jia Jie Woon, Cindy Shuan Ju Teh, Vannajan Sanghiran Lee, Adzzie Shazleen Azman, Sazaly AbuBakar, Hai Yen Lee

**Affiliations:** 1 Tropical Infectious Diseases Research and Education Center (TIDREC), Higher Institution Center of Excellence (HiCOE), Universiti Malaya, Kuala Lumpur 50603, Malaysia; phuichyng.yap@gmail.com (P.-C.Y.); fazlinayuhan@yahoo.com (N.A.); sazaly@um.edu.my (S.A.); 2 Department of Medical Microbiology, Faculty of Medicine, Universiti Malaya, Kuala Lumpur 50603, Malaysia; jiajiewoon93@hotmail.com (J.J.W.); cindysjteh@um.edu.my (C.S.J.T.); 3 Department of Chemistry, Center of Theoretical and Computational Physics (CTCP), Faculty of Science, Universiti Malaya, Kuala Lumpur 50603, Malaysia; vannajan@um.edu.my; 4 School of Science, Monash University Malaysia, Jalan Lagoon Selatan, Bandar Sunway 47500, Malaysia; adzzieshazleen.azman@monash.edu

**Keywords:** infectious diseases, antimicrobial resistance (AMR), lactic acid bacteria, Malaysia, diketopiperazine (DKP)

## Abstract

A total of 20 of isolates of lactic acid bacteria (LAB) were selected and screened for antagonistic activity against clinical strains of 30 clinical isolates of extremely drug-resistant (XDR) *Acinetobacter baumannii* using the well diffusion assay method. Results showed that 50% of the highly LAB strains possessed inhibitory activity against (up to 66%) of the XDR *A. baumannii* strains tested. The supernatant of the twenty LAB strains was subjected to gas chromatography mass spectrometry (GCMS) revealed that the common compound found in the active isolates against XDR *A. baumannii* was 3-Isobutyl-2,3,6,7,8,8a-hexahydropyrrolo[1,2-a]pyrazine-1,4-dione, a known potential diketopiperazine group. The molecular docking study against potential antibacterial targets with selected ligands was performed to predict the binding mode of interactions, which is responsible for antibacterial activity. The docking analysis of the potent compounds supported the potential antibacterial activity exhibiting high inhibition constant and binding affinity in silico.

## 1. Introduction

Lactic acid bacteria (LAB) have been widely studied for their various applications as probiotics and food products. Culture supernatants from LAB were previously characterized for inhibition of bacteria and fungi and further attributed to the production of antifungal peptides in apple [1]. The mechanisms of action of antibacterial activity against other pathogens such as *Shigella sonnei* was due to the production of organic acids and macromolecules involved in inhibition [2]. Studies have also shown that lactic acid bacteria have a vast amount of bioactive compounds that are beneficial as antifungal [3], antibiotic-resistant uropathogens [3], and other human pathogenic bacteria [4], in addition to other potential uses such as anti-cancer, anti-hypertensive, anti-thrombotic, lowering of cholesterol [5], anti-oxidant, and immunomodulation from food proteins [6] in the receptors of the gut epithelium [7]. Further assessment and classification of these bacteria as potential probiotic typically requires in vitro and in vivo study. This study will need to characterize the lactic acid bacteria to survive GI tract conditions, production of antimicrobial substances, tolerance to gastric acid and bile, and ability to adhere or co-aggregate to the intestinal epithelial cells as well as evaluated for the possible transferable antibiotic resistance prior to human clinical trials [8].

The considerable interest in using LAB for the various applications was due to its generally regarded as safe (GRAS) status and as a natural antimicrobial substance. It is also considered as an appropriate alternative for antibiotic treatment and a better pharmaceutical approach [9]. Multidrug resistance (MDR) is a global concern, particularly with extreme and pan drug resistance being reported at an increasing rate in large, highly specialized hospitals [10]. Outbreaks of antimicrobial resistances include *Staphylococcus aureus, Escherichia coli, Klebsiella pneumoniae, Acinetobacter baumannii, Pseudomonas aeruginosa,* or *Enterococcus faecalis* in various hospital settings. *A. baumannii* is the most common and critical nosocomial pathogen of human infection, especially in intensive care units (ICUs) worldwide [11,12,13,14,15]. *A. baumannii* infection is often associated with ventilator-associated pneumonia (VAP), septicemia, meningitis, endocarditis, urinary tract infection (UTI), keratitis, and ophthalmitis, with high morbidity and mortality rates up to 60% [11,13,14]. The antibiotic resistance of *A. baumannii* has gradually developed from multidrug resistance (MDR) to extremely-drug resistance (XDR) [16] and has resulted in several antibiotics such as ureidopenicillin, aminopenicillin, cephalosporin, cefoxitin, chloramphenicol, etc. [12] or all authorized antibiotics except tigecycline and polymyxins [11], showing low efficacy in *A. baumannii* infection treatment. The development of antibiotic resistance is caused by the inactivation of enzymes or the production of enzymes [13], and the resistance genes transfer through plasmids, mutations of targeted genes [14], membrane pore proteins alterations, and active efflux mechanisms, etc. [13,16]. Due to their strong environmental adaptability for a prolonged period [11,16], especially on inanimate objects, this adaptation contributes to their persistence in the medical environment including hand sanitizers, medical personnel belongings, and medical equipment [12]. One of the critical sources of infection is from the biofilm-related contaminated respiratory support devices or suction devices [14] and transmitted via aerosolized *A. baumannii* from infected patients [15]. Although there are still antibiotics available against XDR *A. baumannii* including carbapenems and fluoroquinolones, the minimum inhibitory concentrations (MICs) has gradually increased [12], and some carbapenems-resistant *A. baumannii* has been reported [15,16]. The limited antibiotics against *A. baumannii* show the importance of alternative treatment needed for *A. baumannii* infection. Various strategies are being employed to address MDR including antibiotic stewardship and even redeveloping old antibiotics. *Acinetobacter baumannii* infections have been an ongoing challenge as carbapenems are still the preferred antimicrobial treatment for *Acinetobacter* infections. However, limited study has explored the potential use of lactic acid bacteria against extremely resistant bacteria.

Therefore, the purpose of this study was to screen and identify the potential antagonistic properties of LAB against XDR *Acinetobacter baumannii.*

## 2. Results

### 2.1. Antimicrobial Assay

Twenty active LAB isolates were selected based on the profile of hydrogen peroxide production and tested against *A. baumannii ATCC 19606*, *A. haemolyticus ATCC 19002*, *A. iwoffii ATCC 15309,* and XDR *A. baumannii*. In this antimicrobial assay, findings of the antagonistic activity from twenty of the cell-free supernatants (CFS) from LAB isolates (L001 to L020) were tabulated as Table S1. The isolates were considered active for exhibiting inhibition zones of 10 mm to 12 mm (results not shown). Highly active isolates were found to belong to *Lactobacillus plantarum* and *Pediococcus acidilactici*, for instance, L001 had the highest antagonistic activity up to 66.6% of the XDR *A. baumannii.* The range observed was from 0% of strains susceptible to LAB CFS to as high as 66.6% of the isolates being susceptible. LAB have been widely studied and explored for their potential antimicrobial properties against antibiotic-resistant uropathogens [17] and *Helicobacter pylori* [18]. According to Manzoor, Ul-Haq [17], *L. plantarum* has a remarkable inhibition against the tested multiresistant uropathogens including *Enterococcus faecalis* and *Escherichia coli* up to 29 mm of the inhibition zone, suggesting the potential antibacterial metabolites produced from *L. plantarum* against uropathogens.

### 2.2. Gas Chromatography Mass Spectrometry (GCMS) Analysis 

**Figure 1:**
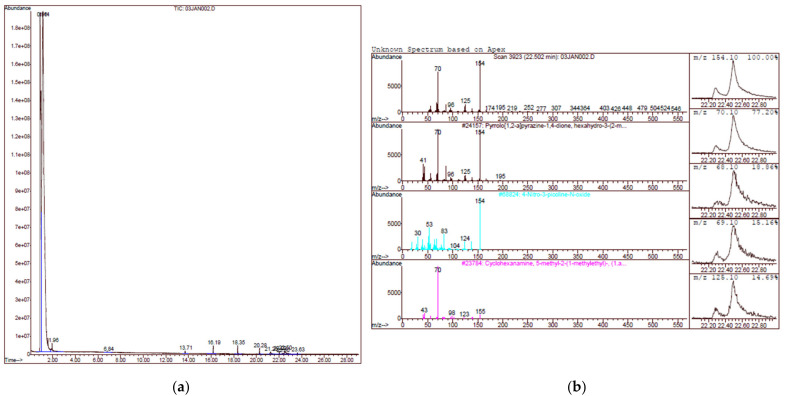
(**a**). GCMS chromatogram of methanolic extract of L001 and (**b**) spectrum analysis of compounds in L001 against pyrolo(1,2-a)pyrazine-1,4-dione.

**Figure 2:**
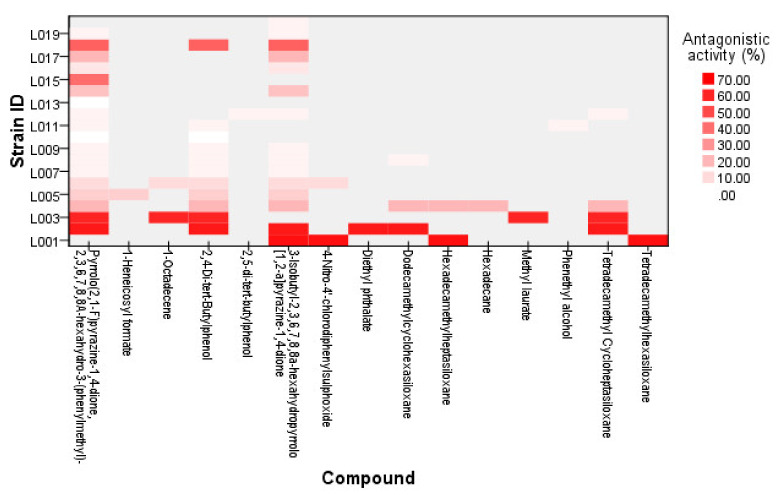
Heat map showing compounds from the lactic acid bacteria strains and antagonistic activities in some XDR *Acinetobacter baumannii* strains (in percentage, *n* = 30).

GCMS analysis of the twenty strains showed that a total of 16 different compounds were detected from the LAB strains (Table 1). Two strains of *Pediococcus* spp. (L010 and L013) did not show any antagonistic activity against all XDR *A. baumannii*, marked as a “0” value, while the highest activity was found in L001 (Figure 1), which was active against 20 out of 30 XDR isolates. A heat map of the compounds and their antagonistic activity value are shown in Figure 2. Three of the most common compounds produced by LAB strains were pyrrolo(2,1-F)pyrazine-1,4-dione,2,3,6,7,8,8A-hexahydro-3-(phenylmethyl)-, 2,4-di-tert-butylphenol, and 3-Isobutyl-2,3,6,7,8,8a-hexahydropyrrolo[1,2-a]pyrazine-1,4-dione. Based on the current finding, the common compound that was found in the strains with antagonistic activity was 3-Isobutyl-2,3,6,7,8,8a-hexahydropyrrolo[1,2-a]pyrazine-1,4-dione and pyrrolo(2,1-F)pyrazine-1,4-dione,2,3,6,7,8,8A-hexahydro-3-(phenylmethyl), which are both cyclodipeptides (CDPs) with proline. When comparing between the three active compounds, it was shown that LAB strains with 0% antagonistic activities did not produce 3-Isobutyl-2,3,6,7,8,8a-hexahydropyrrolo[1,2-apyrazine-1,4-dione while pyrrolo(2,1-F)pyrazine-1,4-dione,2,3,6,7,8,8A-hexahydro-3-(phenylmethyl) showed an antagonistic effect in 16 out of the 18 compound producing LAB strains, suggesting the potential role of CDPs with proline against XDR *A. baumannii* strains.

### 2.3. Molecular Docking and Binding Energy Evaluation

The small ribosomal subunit (30S) fragment and protein structures were selected for interaction with an isolated bioactive compound. One important mechanism of bacterial resistance is the decrease in the permeability of the bacterial membrane. In our investigation for the potential mechanism in bacterial biosynthesis and outer membrane protein, the OmpF proteins complexed with inhibitors, ampicillin (PDB ID: 4GCP), carbenicillin (PDB ID: 4GCQ), and ertapenem (PDB ID: 4GCS) were used to determine the binding interaction with selected compounds. The inhibitors were removed before the docking.

**Figure 3:**
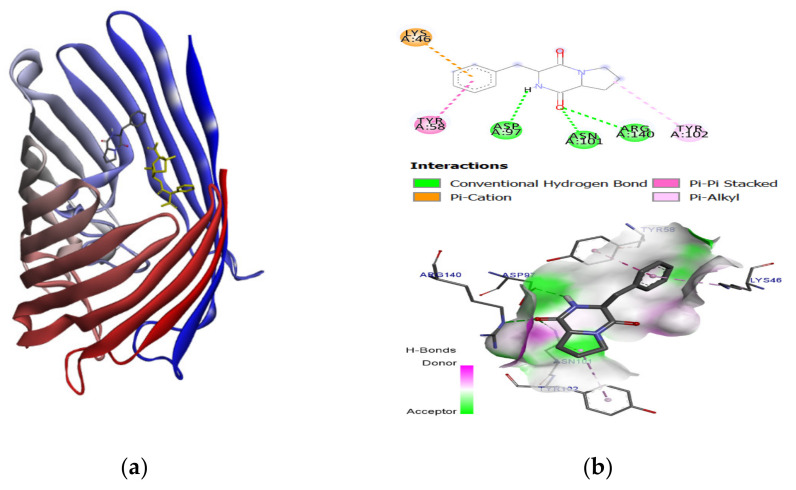
(**a**) In silico interaction of selected ligand pyrrolo (1,2-F) pyrazine-1,4-dione,2,3,6,7,8,8A-hexahydro-3-(phenylmethyl) in stick mode, carbenicillin in yellow and target protein, OmpF porin (4GCQ) (**b**) 2D (top) and 3D level interaction (bottom) of the best-docked complexes illustrate the mode of interaction of the amino acid in the binding pocket. Non-polar hydrogens are not shown.

**Figure 4:**
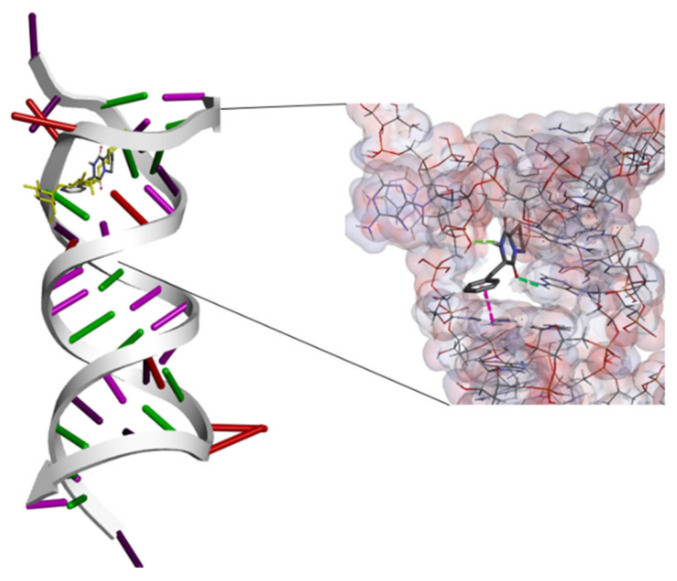
In silico interaction between selected ligand pyrrolo(1,2-F)pyrazine-1,4-d-one,2,3,6,7,8,8A-hexahydro-3-(phenylmethyl) and target RNA fragment (1YRJ). Poses of docked complexes are in the stick model where the x-ray ligand (Paromomycin) in yellow color indicates a prominent active site where the ligand interacted with hydrogen bonding in green and π−π interaction in pink. Non-polar hydrogens are not shown.

In general, to evaluate the prediction of the accuracy of the binding affinity between ligands and target protein from molecular docking, the lower values of binding energy (ΔG) indicate the binding strength of the ligands. The binding energy of ligand-protein docked complexes were analyzed based on minimum binding energy values and the ligand interaction (hydrogen/hydrophobic) pattern. Docking results justified that among the selected candidates, compounds (Pyrrolo(1,2-F)pyrazine-1,4-dione,2,3,6,7,8,8A-hexahydro-3-(phenylmethyl), 4-Nitro-4′-Chlorodiphenylsulphoxide, Dodecamethylcyclohexasiloxane) possessed good binding energy values (−7.1 to −6.0 kcal/mol) compared to the bioactive compound (−8.6 to −6.1 kcal/mol), as mentioned in Table 2. It has been observed that since the structural skeleton of compounds was comparable to the binding pocket; therefore, the binding energy values were also in a similar pattern. In all docking affinity values, the predicted energy values were not higher than 2.5 kcal/mol, indicating that the selected ligands fitted well in the active pocket of the targeted protein. Therefore, the in vitro results were focused on checking their binding profile. The best-ranked pose of the selected compound (Pyrrolo (1,2-F) pyrazine-1,4-dione,2,3,6,7,8,8A-hexahydro-3-(phenylmethyl) was extracted from each binding site and visualized for 2D/3D binding interaction in Figure 3 and Figure 4.

The docking study explored the actual binding pattern within the active site of the target protein. The ligand, pyrrolo(1,2-F)pyrazine-1,4-dione,2,3,6,7,8,8A-hexahydro-3-(phenylmethyl), confined within the same active site with the drug Paromomycin of the target DNA and having a strong binding interaction (−7.0 kcal/mol) with two hydrogens bonding and π−π interaction with the target DNA, is as shown as Figure 3. There is a possibility of this ligand, pyrrolo(1,2-F)pyrazine-1,4-dione,2,3,6,7,8,8A-hexahydro-3-(phenylmethyl) to also have good binding (−6.8 kcal/mol) with the OmpF porin protein (Figure 4). The binding pocket analysis showed that pyrrolo(1,2-F)pyrazine-1,4-dione,2,3,6,7,8,8A-hexahydro-3-(phenylmethyl) binds with several amino acids, Lys46, Tyr58, Asp97, Asn101, Tyr102, and Arg140, in the binding region of the target protein. It has been observed that three hydrogen bonds were observed at Asp97, Asn101, and Arg140 with 14705-60-3 and π−π, π−cation, and π−π-alkyl interaction were found. Our results justified that pyrrolo(1,2-F)pyrazine-1,4-dione,2,3,6,7,8,8A-hexahydro-3-(phenylmethyl) firmly binds to both bacterial membranes with active binding residues of the OmpF porin protein and 16S rRNA, which could functionally participate in the inhibition of DNA synthesis.

## 3. Discussion

The clinical strains of *A. baumannii* used in this study are extremely drug-resistant, especially to β-lactam (AP, SAM, AMC, CXM, CRO, CTX, IPM, MEM, FEP, and TZP) and fluoroquinolone (CIP) antibiotics, which are active against all strains. The β-lactam antibiotic is known to interfere with the synthesis of bacterial cell wall through irreversible transpeptidase enzyme inhibition whereas fluoroquinolone inhibits DNA replication through DNA gyrase interaction [19]. Porin channel and outer membrane protein (OMP) embedded in the microbial membrane allow for the exchange of molecules including antibiotics between intracellular and outer space. Downregulation of OMP gene expression increased antibiotic-resistant of *A. baumannii* as the access of the antibiotic to intracellular was reduced, which plays an important role in resistance mechanisms. The presence of β-lactam in periplasmic space triggers *A. baumannii* to possess an enzymatic reaction of β-lactamases such as *Acinetobacter-*derived cephalosporinases (ADCs) through AmpC gene expression, which is involved in the hydrolysis of β-lactam such as IPM, MEM, and FEP, or efflux pump to eliminate antibiotics from cell cytoplasm [20,21]. Production of CDPs by LAB has been shown to potentiate the inhibition of XDR *A. baumannii.* The majority of CDPs are produced as secondary metabolites by bacteria and are considered as the smallest possible CDPs among cyclic organic compounds in diketopiperazines (DKPs), which consist of two nitrogen atoms of a piperazine 6-membered ring forming an amide bridge [22]. The stable and rigid structure, chiral nature, protease resistance, and functionalized structures of CDPs make them possible to bind to the various receptors with high affinity as reported in 3-Isobutyl-2,3,6,7,8,8a-hexahydropyrrolo[1,2-a]pyrazine-1,4-dione (−5.5 kcal/mol) and Pyrrolo(2,1-F)pyrazine-1,4-dione,2,3,6,7,8,8A-hexahydro-3-(phenylmethyl) (−6.8 kcal/mol). The high binding of CDPs to microbial membrane receptors such as OMP exerts a radical scavenging effect and an overall antagonistic effect against various biological cells including pathogenic bacteria, viruses, fungi, and tumors. These properties predominate as an attractive model for drug design [22,23,24]. The inhibition mechanism of CDPs could be due to their hydrophobic nature, which could interfere with the bacterial cell membrane, influencing their function and loss of cell integrity, causing cell death [25]. Interestingly, the high binding of CDPs with 16S rRNA was observed in this study (up to −7.0 kcal/mol), suggesting the possibility of transportation of CDPs into intracellular of XDR *A. baumannii* and further binding to 16S rRNA and interrupting DNA synthesis. Findings in the current study may be supported by the fact that CDPs are biosynthesized by LAB, which is believed to involve protease enzymes that cleave terminal ends of amino acids, creating dipeptides and cyclize naturally involving enzymes such as non-ribosomal peptide synthetases (NRPSs) and CDPs synthases [22]. The compound, 3-Isobutyl-2,3,6,7,8,8a-hexahydropyrrolo[1,2-a]pyrazine-1,4-dione is also known as cyclo (d-Pro-l-Leu) or cyclo (l-Pro-d-Leu). Antimicrobial activity of 3-Isobutyl-2,3,6,7,8,8a-hexahydropyrrolo[1,2-a]pyrazine-1,4-dione has been widely reported against *Candida albicans* with minimum inhibitory concentration (MIC) of 16 μg/mL and minimum fungicidal concentration (MFC) of 32 μg/mL [24]; *Aspergillus flavus* and *Aspergillus niger* with MIC of 8 μg/mL and 32 μg/mL, respectively, which are 500-fold and 156-fold lower compared to the control [26]; *Mycobacterium tuberculosis* with MIC of 8 μg/mL and minimum bactericidal concentration (MBC) of 16 μg/mL [23]; *Vibro anguillarum* with MIC of 0.13 μg/mL, which was about four times lower than oxytetracycline (0.5 μg/mL) [27]. Pyrrolo(2,1-F)pyrazine-1,4-d-one,2,3,6,7,8,8A-hexahydro-3-(phenylmethyl)- is also known as cyclo (d-Phe-l-Pro). Cyclo (l-Pro-d-Phe) obtained from *Streptomyces* sp. DA18 shown moderate antimicrobial activity against *Escherichia coli*, *Bacillus subtilis*, *Pseudomonas fluorescens,* and *Candida albican* [28]. Carvalho and Abraham (2012) reported the potential of cyclo (d-Phe-l-Pro) in exerting moderate inhibitory activity against *V. anguillarum* with a MIC of 0.13 μg/mL compared to its isomer, cyclo (d-Phe-d-Pro) with a MIC of 0.03 μg/mL [29]. Despite their isomerization, Liu et al. (2017) reported that the proline-based CDPs had significant antagonistic activity in bacteria [30]. For example, cyclo (Leu-Pro)^a^ and cyclo (Phe-Pro)^b^ produced by *Lactobacillus plantarum* LBP-K10 have antibacterial activity against Gram-positive such as *B. subtilis* (MIC: 13.55 μg/mL^a^ and 45.88 μg/mL^b^) and MDR *S. aureus* 11471 (MIC: 17.28 μg/mL^a^ and 46.22 μg/mL^b^) and Gram-negative such as *E. coli* (MIC: 10.41 μg/mL^a^ and 35.68 μg/mL^b^) and MDR *Salmonella typhimurium* 12219 (MIC: 18.19 μg/mL^a^ and 43.70 μg/mL^b^). Confirmation of CDP’s role in the inhibition of XDR strains still require further investigation; however, the preliminary results showed that inhibition was apparent in this study.

Another exciting compound identified in this study was 2,4-di-tert-butylphenol(2,4 DTBP), which was detected in 11 out of 20 LAB strains. All strains showed antagonistic activity against at least 10% up to 70% of the XDR strains, except that L010 showed no effect. This could be due to the concentration of this compound produced did not reach the threshold to exert the effect, which also applied to L013 or due to the presence of some other compounds that interfere with 2,4 DTBP activity. Lertcanawa et al. (2016) reported that the combination of vancomycin and 2,4 DTBP led to inactivation of antagonistic activity against methicillin-resistant *Staphylococcus aureus* (MRSA) due to enzyme catalyzation that contributes to the formation of other compounds that may have no effect opposing MRSA, and eventually reduce the concentration of 2,4 DTBP [31]. On the other hand, 2,4 DTBP obtained from *Streptomyces* species has reported exerting a similar effect as vancomycin in MRSA by cell deformation, with a MIC of 31.25 μg/mL and 1.56 μg/mL, which are much lower compared to oxacillin (>100.00 μg/mL) [32]. The mechanism action of 2,4 DTBP against MRSA is found through their penetration into the cell membrane and interfere with the cell integrity and permeability by interaction with the hydrophobic tails of the phospholipid bilayer, causing cytolysis and the release of genetic material, hence cell death [31]. According to Aissaoui et al. (2018), thermophilic bacteria isolates were found to produce ~15% of 2,4 DTBP, which was identified as the essential compound to inhibit MDR bacteria including *Pseudomonas aeruginosa* and *Staphylococcus aureus* [33]. The compound 2,4 DTBP was also shown to have antibiofilm formation of pathogenic bacteria, *Streptococcus pyogenes* up to 79% inhibition at a 48 μg/mL concentration [34].

The molecular docking studies buoyed that selected compounds can work as a lead inhibitor of bacterial DNA synthesis and membrane permeation due to conformational fitting in the active site of the targeted protein, especially compound pyrrolo (2,1-F) pyrazine-1,4-dione,2,3,6,7,8,8A-hexahydro-3-(phenylmethyl)-(14705-60-3), which has a greater binding affinity (−6.4 kcal/mol) compared to antibiotic Ertapenem (−6.1 kcal/mol). While there are many in silico models and detection of compounds against MDR and XDR tuberculosis [35,36], limited studies have reported active compounds and in silico docking against XDR *A. baumannii*. Therefore, to the best of our knowledge, this is the first report on the identification of DKP against XDR *A. baumannii.*

## 4. Materials and Methods 

### 4.1. Approval by Institutional Biosafety and Biosecurity Council and Ethics Committee

Approval for this project was obtained from Institutional Biosafety and Biosecurity Council University Malaysia (UMIBBC/NOI/R/FOM/TIDREC-003/2017-rev01) for the work with potentially infectious materials. Collection of clinical bacterial strains and secondary data such as patients’ demographic and clinical data were conducted under the approval of Universiti Malaya Medical Center (UMMC) Medical Research Ethics Committee (MREC) (IRB Reference number: 1073.21). The UMMC MREC did not require the written informed consent from participants because the isolates were obtained from the hospital’s diagnostic lab, and patients were not directly involved in this study.

### 4.2. Sampling and Isolation of Lactic Acid Bacteria

A total number of 150 samples from food sources and the environment was collected for the sampling process using the modified method from Monika, Savitri [37]. In brief, 10 g of samples were diluted in 90 mL of MRS broth for LAB. Samples were then homogenized and incubated for 24–48 h using the anaerobic condition at 37 °C. Samples were then streaked onto MRS agar and further incubated up to 48 h to obtain colonies. Presumptive colonies were isolated and purified for identification using the molecular method.

### 4.3. Molecular Identification and Hydrogen Peroxide Production

Pure colonies were obtained from streaking and resuspended in 500 µL of distilled water. DNA extraction was performed using the boil cell method [38]; in brief, the suspension was incubated in the dry block at 100 °C for 20 min and immediately transferred to freezing at −20 °C for 10 min. Tubes were then centrifuged under 12,000 rpm for 2 min, and the supernatant was used as a DNA template.

LAB isolated from food samples were identified using 16S rRNA primers 27F (5′-AGAGTTTGATCCTGGCTCAG-3′) and 1492R2 (5′-TACGGYTACCTTGTTACGACTT-3′) described in [39]. The polymerase chain reaction mix using GoTaq^®^ Green Polymerase reaction consisted of 1x colorless buffer, 3 mM of MgCl_2_, 2.5 mM of dNTP mix, 300 pmol of primers, 1.5 U Taq Polymerase, and 3 uL of template DNA. Gel electrophoresis was performed at 100 V for 40 min in 1% agarose and further proceeded for sequencing services.

Isolates were also screened for hydrogen peroxide production using the Prussian-blue agar method described in Saito, Seki [40], and were characterized based on light blue changes in the agar.

### 4.4. Well-Diffusion Antimicrobial Assay

Evaluation of antimicrobial activity was conducted using the well diffusion method described in Hoover and Harlander [41]. Each LAB colony was purified to obtain a single colony, and glycerol stock was prepared. Glycerol stock was used to inoculate MRS broth for *Lactobacillus* bacteria. The estimation of cell count was based on the McFarland standard and OD600 reading to obtain 10^10^ CFU/mL using an appropriate blank control.

LAB cultures were then centrifuged at 12,000× *g* 5 min, and the supernatant was treated to exclude the inhibitory effect of other compounds including organic acids by adjusting to pH 6.0–6.5 with 5 N NaOH and hydrogen peroxide by adding catalase to a final concentration of 1 mg/mL. The cell-free supernatant (CFS) was subjected to filter sterilization using a 0.2 µm syringe filter and transferred into the new sterile tube for analysis. A total of 395 CFS were screened for antibacterial activity against *Acinetobacter baumannii* ATCC 19606, *Acinetobacter haemolyticus* ATCC 19002, and *Acinetobacter iwoffii* ATCC 15309. Control strains were grown on Mueller Hinton broth and adjusted to 10^8^ CFU/mL and seeded onto Mueller Hinton Agar. Agar wells were punctured to create a 5 mm diameter well. The wells were loaded with 10 µL of the filter-sterilized CFS from lactic acid bacteria. Agar plates were then incubated at 37 °C for 24 h, and results were interpreted as ‘positive’ or ‘negative’. Any zone of inhibition above 1 mm was considered as ‘positive’ and selected to further evaluation.

### 4.5. Screening Against Clinical Isolates of Extremely Drug Resistance (XDR) Acinetobacter baumannii

The twenty most active strains of lactic acid bacteria with low hydrogen peroxide production were selected to reduce the possibility of inhibition resulting from hydrogen peroxide and tested against 30 strains of XDR *A. baumannii*. Clinical isolates of XDR *A. baumannii* were obtained from the University Malaya Medical Center (UMMC). XDR strains were grown in TSB and adjusted to a concentration of 10^6^ CFU/mL for well diffusion disc agar antimicrobial assay, as described above. Agar plates were then incubated at 37 °C for 24 h, and results were interpreted as ‘positive’ or ‘negative’. Antibiotic resistance profile of XDR *A. baumannii* was tabulated as Table S2.

### 4.6. GCMS 

The selected twenty strains of LAB (L001–L020) were cultured in 100 mL of MRS broth (Himedia, India) in a CO_2_ incubator and adjusted to 10^10^ CFU/mL using a spectrophotometer at 600 nm. Cultures were centrifuged at 5000× *g* for 10 min, and the cell-free supernatant was collected. The supernatant was partially characterized as described above, and freeze-dried, followed by the addition of an equal volume of methanol and allowed to stand for 48 h and subjected to the rotary evaporator for methanol extraction at 50 °C for 1 h. The compounds were collected for GCMS analysis using a HP–5 column, 0.32 mm × 30 mm × 0.25 µm nominal with helium as the carrier gas at a flow rate at 7.7 mL/min. The interpretation on the mass spectrum from GCMS was performed using the database of the National Institute Standard and Technology (NIST) 98. The mass spectrum of the compounds detected from the methanolic extract was compared with the spectrum of known compounds stored in the NIST library to determine the name, molecular weight, and structure of the compound. The determination of the identity of the compound was set to be at above 80% for the quality of signal and identity.

### 4.7. In Silico Investigation toward Antimicrobial Multi-Protein Targets

To elucidate the potential mechanism, selected target proteins in bacterial biosynthesis and outer-membrane protein F (OmpF) porin were accessed from the Protein Data Bank (PDB) (www.rcsb.org (accessed on 18 July 2020)) as follows:

- 4GCP: Crystal structure of *E. coli* OmpF porin in complex with ampicillin
- 4GCQ: Crystal structure of *E. coli* OmpF porin in complex with carbenicillin
- 4GCS: Crystal structure of *E. coli* OmpF porin in complex with ertapenem
- 1YRJ: Crystal structure of apramycin bound to a ribosomal RNA
- 1MWL: Crystal structure of geneticin bound to the eubacterial 16S rRNA
- 1J7T: Crystal structure of paromomycin and the 16S rRNA
- 1LC4: Crystal structure of tobramycin bound to the eubacterial 16S rRNA

The selected target proteins were minimized with the Amber force field by employing a conjugate gradient algorithm in UCSF Chimera 1.10.1 [42]. All protein structures were prepared for docking using the protein preparation in Chimera software with the default protocol for PDB2PQR and Dock Prep. Protonation state was assigned using PROPKA at pH 7.0, and gasteiger charges were assigned for protein. Selected ligands were downloaded from PubChem and optimized using the AM1 level using the Gaussian software package. Molecular docking was performed with a local search algorithm using Autodock Vina in PyRx virtual screening software (http://pyrx.scripps.edu/ (accessed on 18 July 2020) considered the target conformation as a rigid unit while the ligands were allowed to be flexible and adaptable to the target. The amino acid binding sites were selected at the ligand center from the X-ray structure. The grid box was set at 20 × 20 × 20 Å^3^ with the default grid spacing of 0.375. The 2D and 3D interactions were visualized by BIOVIA Discovery Studio software [43] for different conformations for each ligand and the lowest binding affinity conformations were predicted.

## 5. Conclusions

LAB are prospective antibiotic replacements due to their ability to produce CDPs as metabolites, namely 3-Isobutyl-2,3,6,7,8,8a-hexahydropyrrolo[1,2-a]pyrazine-1,4-dione and pyrrolo (2,1-F)pyrazine-1,4-dione,2,3,6,7,8,8A-hexahydro-3-(phenylmethyl). These compounds potentiate the inactivation of XDR *A. baumanii* clinical strains through their highly binding affinity to *A. baumannii* membrane receptors and intracellular 16S rRNA (−5.4 to −7.1 kcal/mol). However, the mechanisms of DKPs and CDPs in microbial inactivation should be further investigated and their potential in antibiotic stewardship by the reduction of antibiotic utilization, hence combating antibiotic-resistance.

## Figures and Tables

**Table 1:** List of compounds identified from lactic acid bacteria strains and their antagonistic activity against 30 extremely drug resistance (XDR) *Acinetobacter baumannii* isolates.

Sample ID	Antagonistic Activity in 30 XDR *A. Baumannii* Strains (%)	CAS NO.	Compound ID
L001 (*Lactobacillus plantarum*)	20/30 (66.0)	000541-01-5	Hexadecamethylheptasiloxane
		005654-86-4	3-Isobutyl-2,3,6,7,8,8a-hexahydropyrrolo[1,2-a]pyrazine-1,4-dione
		024535-53-3	4-Nitro-4′-chlorodiphenylsulfoxide
		000107-52-8	Tetradecamethylhexasiloxane
L002 (*Lactobacillus plantarum*)	19/30 (63.3)	000084-66-2	Diethyl phthalate
		000096-76-4	2,4-Di-tert-Butylphenol
		000540-97-6	Dodecamethylcyclohexasiloxane
		005654-86-4	3-Isobutyl-2,3,6,7,8,8a-hexahydropyrrolo[1,2-a]pyrazine-1,4-dione
		014705-60-3	Pyrrolo(2,1-F)pyrazine-1,4-dione,2,3,6,7,8,8A-hexahydro-3-(phenylmethyl)-
		000107-50-6	Tetradecamethyl Cycloheptasiloxane
L003 (*Lactobacillus plantarum*)	18/30 (60.0)	000096-76-4	2,4-Di-tert-Butylphenol
		000112-88-9	1-Octadecene
		000111-82-0	Methyl laurate
		014705-60-3	Pyrrolo(2,1-F)pyrazine-1,4-dione,2,3,6,7,8,8A-hexahydro-3-(phenylmethyl)-
		000107-50-6	Tetradecamethyl Cycloheptasiloxane
L004 (*Pediococcus pentosaceus*)	5/30 (16.7)	000096-76-4	2,4-Di-tert-Butylphenol
		000540-97-6	Dodecamethylcyclohexasiloxane
		000541-01-5	Hexadecamethylheptasiloxane
		005654-86-4	3-Isobutyl-2,3,6,7,8,8a-hexahydropyrrolo[1,2-a]pyrazine-1,4-dione
		000544-76-3	Hexadecane
		014705-60-3	Pyrrolo(2,1-F)pyrazine-1,4-dione,2,3,6,7,8,8A-hexahydro-3-(phenylmethyl)-
		000107-50-6	Tetradecamethyl Cycloheptasiloxane
L005 (*Pediococcus pentosaceus*)	4/30 (13.3)	000096-76-4	2,4-Di-tert-Butylphenol
		005654-86-4	3-Isobutyl-2,3,6,7,8,8a-hexahydropyrrolo[1,2-a]pyrazine-1,4-dione
		077899-03-7	1-Heneicosyl formate
		014705-60-3	Pyrrolo (2,1-F) pyrazine-1,4-dione,2,3,6,7,8,8A-hexahydro-3-(phenylmethyl)-
L006 (*Pediococcus pentosaceus*)	3/30 (10.0)	000096-76-4	2,4-Di-tert-Butylphenol
		000112-88-9	1-Octadecene
		005654-86-4	3-Isobutyl-2,3,6,7,8,8a-hexahydropyrrolo[1,2-a]pyrazine-1,4-dione
		024535-53-3	4-Nitro-4′-chlorodiphenylsulphoxide
		014705-60-3	Pyrrolo(2,1-F)pyrazine-1,4-dione,2,3,6,7,8,8A-hexahydro-3-(phenylmethyl)
L007 (*Pediococcus pentosaceus*)	1/30 (3.3)	000096-76-4	2,4-Di-tert-Butylphenol
		005654-86-4	3-Isobutyl-2,3,6,7,8,8a-hexahydropyrrolo[1,2-a]pyrazine-1,4-dione
		014705-60-3	Pyrrolo (2,1-F) pyrazine-1,4-dione,2,3,6,7,8,8A-hexahydro-3-(phenylmethyl)
L008 (*Pediococcus pentosaceus*)	1/30 (3.3)	000096-76-4	2,4-Di-tert-Butylphenol
		000540-97-6	Dodecamethylcyclohexasiloxane
		005654-86-4	3-Isobutyl-2,3,6,7,8,8a-hexahydropyrrolo [1,2-a] pyrazine-1,4-dione
		014705-60-3	Pyrrolo(2,1-F)pyrazine-1,4-dione,2,3,6,7,8,8A-hexahydro-3-(phenylmethyl)
L009 (*Pediococcus acidilactici*)	1/30 (3.3)	000096-76-4	2,4-Di-tert-Butylphenol
		005654-86-4	3-Isobutyl-2,3,6,7,8,8a-hexahydropyrrolo[1,2-a]pyrazine-1,4-dione
		014705-60-3	Pyrrolo(2,1-F)pyrazine-1,4-dione,2,3,6,7,8,8A-hexahydro-3-(phenylmethyl)-
L010 (*Pediococcus* spp.)	0/30 (0)	000096-76-4	2,4-Di-tert-Butylphenol
		014705-60-3	Pyrrolo(2,1-F)pyrazine-1,4-dione,2,3,6,7,8,8A-hexahydro-3-(phenylmethyl)-
L011 (*Pediococcus acidilactici*)	1/30 (3.3)	000096-76-4	2,4-Di-tert-Butylphenol
		000060-12-8	Phenethyl alcohol
		014705-60-3	Pyrrolo(2,1-F)pyrazine-1,4-dione,2,3,6,7,8,8A-hexahydro-3-(phenylmethyl)
L012 (*Pediococcus pentosaceus*)	1/30 (3.3)	005654-86-4	3-Isobutyl-2,3,6,7,8,8a-hexahydropyrrolo[1,2-a]pyrazine-1,4-dione
		005875-45-6	2,5-di-tert-butylphenol
		014705-60-3	Pyrrolo(2,1-F)pyrazine-1,4-dione,2,3,6,7,8,8A-hexahydro-3-(phenylmethyl)
		000107-50-6	Tetradecamethyl Cycloheptasiloxane
L013 (*Pediococcus pentosaceus*)	0/30 (0)	014705-60-3	Pyrrolo(2,1-F)pyrazine-1,4-dione,2,3,6,7,8,8A-hexahydro-3-(phenylmethyl)
L014 (*Pediococcus pentosaceus*)	5/30 (16.7)	005654-86-4	3-Isobutyl-2,3,6,7,8,8a-hexahydropyrrolo[1,2-a]pyrazine-1,4-dione
		014705-60-3	Pyrrolo(2,1-F)pyrazine-1,4-dione,2,3,6,7,8,8A-hexahydro-3-(phenylmethyl)-
L015 (*Pediococcus pentosaceus*)	12/30 (40.0)	014705-60-3	Pyrrolo(2,1-F)pyrazine-1,4-dione,2,3,6,7,8,8A-hexahydro-3-(phenylmethyl)-
L016 (*Enterococcus* spp.)	2/30 (6.7)	005654-86-4	3-Isobutyl-2,3,6,7,8,8a-hexahydropyrrolo[1,2-a]pyrazine-1,4-dione
		014705-60-3	Pyrrolo(2,1-F)pyrazine-1,4-dione,2,3,6,7,8,8A-hexahydro-3-(phenylmethyl)
L017 (*Pediococcus acidilactici*)	6/30 (20.0)	005654-86-4	3-Isobutyl-2,3,6,7,8,8a-hexahydropyrrolo[1,2-a]pyrazine-1,4-dione
		014705-60-3	Pyrrolo (2,1-F) pyrazine-1,4-dione,2,3,6,7,8,8A-hexahydro-3-(phenylmethyl)
L018 (*Pediococcus pentosaceus*)	13/20 (43.3)	000096-76-4	2,4-Di-tert-Butylphenol
		005654-86-4	3-Isobutyl-2,3,6,7,8,8a-hexahydropyrrolo [1,2-a] pyrazine-1,4-dione
		014705-60-3	Pyrrolo (2,1-F) pyrazine-1,4-dione,2,3,6,7,8,8A-hexahydro-3-(phenylmethyl)
L019 (*Lactobacillus paraplantarum*)	1/30 (3.3)	005654-86-4	3-Isobutyl-2,3,6,7,8,8a-hexahydropyrrolo [1,2-a] pyrazine-1,4-dione
		014705-60-3	Pyrrolo (2,1-F) pyrazine-1,4-dione,2,3,6,7,8,8A-hexahydro-3-(phenylmethyl)
L020 (*Enterococcus* spp.)	1/30 (3.3)	005654-86-4	3-Isobutyl-2,3,6,7,8,8a-hexahydropyrrolo [1,2-a] pyrazine-1,4-dione

**Table 2:** Docking energy values (ΔG in kcal/mol) of the selected compound. The highlighted compounds were bound well over the selected investigation with the targets.

Compound ID (CAS NO.)	Autodock Vina Binding Affinity (kcal/mol)						
	OmpF Porin Protein			16S rRNA			
	4GCP	4GCQ	4GCS	1YRJ	1MWL	1J7T	1LC4
Tetradecamethylhexasiloxane (107-52-8 Si)	−4.8	−4.6	−4.5	−6.3	−5.6	−5.9	−5.3
Dodecamethylcyclohexasiloxane (540-97-6 Si)	−5.8	−6.4	−5.6	−6.0	−6.2	−6.1	−6.6
Hexadecamethylheptasiloxane (541-01-5 Si)	−5.0	-4.9	−4.2	−6.1	−5.3	−5.8	−6.2
3-Isobutyl-2,3,6,7,8,8a-hexahydropyrrolo[1,2-A] Pyrazine-1,4-Dione (5654-86-4)	−5.4	−5.4	−5.5	−6.1	−5.7	−6.0	−5.9
4-Nitro-4′-Chlorodiphenylsulphoxide (24535-53-3)	−6.1	−6.7	−6.1	−6.4	−6.6	−6.5	−6.3
Diethyl Phthalate (84-66-2)	−5.3	−5.6	−5.1	−5.3	−5.7	−5.8	−5.3
2,4-Di-Tert-Butylphenol (96-76-4)	−5.9	−6.0	−5.9	−5.3	−5.1	−5.7	−5.2
Pyrrolo (1,2-F) pyrazine-1,4-dione,2,3,6,7,8,8A-hexahydro-3-(phenylmethyl) (14705-60-3)	−6.4	−6.8	−6.4	−7.1	−6.5	−7.0	−6.5
1-Octadecene (112-88-9)	−4.2	−4.9	−4.1	−3.6	−3.2	−4.0	−3.4
Hexadecane (544-76-3)	−4.1	−4.6	−4.7	−3.5	−3.2	−3.5	−3.0
PhenethylAlcohol (60-12-8)	−4.3	−4.9	−4.3	−4.0	−4.3	−4.4	−4.2
1-Heneicosylformate (77899-03-7)	−4.5	−4.2	−4.2	−4.0	−3.9	−4.4	−3.9
Methyl laurate (111-82-0)	−4.5	−4.7	−4.7	−4.1	−3.7	−4.2	−3.7
2,5-di-tert-butylphenol (5875-45-6)	−5.8	−6.0	−5.8	−5.7	−5.3	−5.6	−5.6
Xray Ligand	Ampicillin	Carbenicillin	Ertapenem	Apramycin	Geneticin	Paromomycin	Tobramycin
	−7.1	−7.1	−6.1	−8.2	−7.2	−8.6	−7.6

## Data Availability

All data generated and analyzed during this study are included in this manuscript.

## Supplementary Materials

The following are available online, Table S1: Identification of lactic acid bacteria strains selected for further screening. Table S2: Antibiotic resistance profile of XDR *A. baumannii* strains tested in this study.
