# Supplementary material for: Profiling of Potential Antibacterial Compounds of Lactic Acid Bacteria against Extremely Drug Resistant (XDR) Acinetobacter baumannii
Source: Molecules. 2021 Mar 19;26(6):1727. doi: 10.3390/molecules26061727 (PMC8003687; doi:10.3390/molecules26061727)
Supplement: Supplementary file 1 [file molecules-26-01727-s001.zip › molecules-1090991-supplementary.pdf]

Table S1: Identification of lactic acid bacteria strains selected for further screening

ID Isolates No	Identity of isolates	Source	Pathogens (Control strains)			Pathogens (30 clinical strains of *A. baumannii*)																													
			A. baumannii	*A. haemolyticus*	*A. lwoffii*	AB 11	AB 12	AB 13	AB 14	AB 15	AB 16	AB 17	AB 18	AB 19	AB 20	AB 21	AB 22	AB 23	AB 24	AB 25	AB 26	AB 27	AB 28	AB 29	AB 30	AB 31	AB 32	AB 33	AB 34	AB 35	AB 36	AB 37	AB 38	AB 39	AB 40
L001	*Lactobacillus plantarum*	Fermented cabbage	✓	✓	✓	✓		✓	✓				✓	✓			✓	✓	✓		✓	✓		✓		✓		✓	✓	✓	✓	✓	✓	✓	✓
L002	*Lactobacillus plantarum*	Fermented cabbage	✓	✓	✓			✓	✓				✓	✓			✓	✓		✓	✓	✓		✓		✓		✓	✓	✓	✓	✓	✓	✓	✓
L003	*Lactobacillus plantarum*	Fermented cabbage	✓		✓		✓	✓	✓			✓			✓	✓	✓	✓			✓			✓				✓	✓	✓	✓	✓	✓	✓	✓
L004	*Pediococcus pentosaceus*	Cherry pickle	✓	✓	✓			✓												✓	✓				✓								✓		
L005	*Pediococcus pentosaceus*	Cherry pickle	✓	✓	✓			✓										✓		✓					✓										
L006	*Pediococcus pentosaceus*	Hog plum pickle	✓	✓	✓															✓	✓				✓										

L007	*Pediococcus pentosaceus*	Hog plum pickle	✓	✓	✓																			✓											
L008	*Pediococcus pentosaceus*	Hog plum pickle	✓	✓	✓														✓																
L009	*Pediococcus acidilactici*	Green mango pickle	✓	✓	✓																														✓
L010	*Pediococcus spp.*	Green mango pickle																																	
L011	*Pediococcus acidilactici*	Yogurt	✓	✓	✓																			✓											
L012	*Pediococcus pentosaceus*	FYogurt	✓	✓	✓																			✓											
L013	*Pediococcus pentosaceus*	Pickled turnip																																	
L014	*Pediococcus pentosaceus*	Fermented chilli	✓					✓													✓		✓						✓		✓				

L015	*Pediococcus pentosaceus*	Soil	✓	✓	✓		✓	✓									✓	✓			✓						✓	✓	✓	✓		✓	✓		✓
L016	*Enterococcus spp.*	Soil		✓	✓			✓										✓																	
L017	*Pediococcus acidilactici*	Fermented cabbage	✓	✓				✓										✓			✓	✓	✓										✓		
L018	*Pediococcus pentosaceus*	Fermented glutinious rice	✓	✓	✓	✓		✓			✓	✓	✓				✓	✓					✓		✓			✓	✓		✓		✓		
L019	*Lactobacillus paraplantarum*	Raw fresh milk	✓		✓																											✓			
L020	*Enterococcus spp.*	Ready-to-eat milk	✓																													✓			

Note: "✓" indicating active against selected pathogen with zone of inhibition between 10mm to 12mm.

Table S2. Antibiotic resistance profile of XDR *A. baumannii* strains tested in this study

Isolates	Source	Antimicrobial Susceptibility Profile																		
		AP	SXT	SAM	AMC	CN	NET	CXM	CAZ	CRO	CTX	PIP	CIP	IPM	MEM	FEP	SCF	TZP	AN	CT
Ab 11	Tracheal Aspirate	R	S	R	R	R	R	R	R	R	R	R	R	R	R	R	R	R	R	S
Ab 12	Tracheal Secretion	R	R	R	R	R	R	R	R	R	R	R	R	R	R	R	R	R	R	S
Ab 13	Tracheal Secretion	R	R	R	R	R	R	R	R	R	R	R	R	R	R	R	R	R	R	S
Ab 14	Tracheal Aspirate	R	R	R	R	R	R	R	R	R	R	R	R	R	R	R	R	R	R	S
Ab 15	Tracheal Aspirate	R	S	R	R	S	S	R	R	R	R	R	R	R	R	R	R	R	S	S
Ab 16	Skin Blister	R	S	R	R	R	R	R	R	R	R	R	R	R	R	R	R	R	S	S
Ab 17	Blood	R	R	R	R	R	R	R	R	R	R	R	R	R	R	R	R	R	R	S
Ab 18	Tracheal Aspirate	R	S	R	R	R	R	R	R	R	R	R	R	R	R	R	R	R	R	S
Ab 19	Tracheal Aspirate	R	R	R	R	R	R	R	R	R	R	R	R	R	R	R	R	R	S	S
Ab 20	Tracheal Secretion	R	S	R	R	R	R	R	R	R	R	R	R	R	R	R	R	R	R	S
Ab 21	Tracheal Aspirate	R	R	R	R	R	R	R	R	R	R	R	R	R	R	R	R	R	R	S
Ab 22	Tracheal Secretion	R	R	R	R	R	R	R	R	R	R	R	R	R	R	R	R	R	R	S
Ab 23	Tracheal Aspirate	R	S	R	R	S	S	R	R	R	R	R	R	R	R	R	IN	R	S	S
Ab 24	Flank tissue	R	R	R	R	R	R	R	R	R	R	R	R	R	R	R	R	R	R	S
Ab 25	Tracheal Aspirate	R	R	R	R	R	R	R	R	R	R	R	R	R	R	R	R	R	R	S
Ab 26	Blood	R	R	R	R	R	R	R	R	R	R	R	R	R	R	R	R	R	R	S
Ab 27	Tracheal Secretion	R	R	R	R	R	R	R	R	R	R	R	R	R	R	R	R	R	R	S
Ab 28	Sputum	R	S	R	R	S	R	R	R	R	R	R	R	R	R	R	R	R	S	S
Ab 29	Blood	R	R	R	R	R	R	R	R	R	R	R	R	R	R	R	R	R	R	S
Ab 30	Tracheal Secretion	R	R	R	R	R	R	R	R	R	R	R	R	R	R	R	S	R	R	S
Ab 31	Sputum	R	R	R	R	R	R	R	R	R	R	R	R	R	R	R	R	R	R	S
Ab 32	Sputum	R	R	R	R	R	R	R	R	R	R	R	R	R	R	R	R	R	R	S

Ab 33	Thigh Swab	R	S	R	R	IN	R	R	R	R	R	R	R	R	R	R	R	R	R	S
Ab 34	Tracheal Secretion	R	R	R	R	R	R	R	R	R	R	R	R	R	R	R	R	R	R	S
Ab 35	Brochoalveolar Lavage	R	R	R	R	IN	R	R	R	R	R	R	R	R	R	R	R	R	R	S
Ab 36	Tracheal Secretion	R	R	R	R	R	-	R	R	R	R	-	R	R	R	R	S	R	R	S
Ab 37	Tracheal Secretion	R	R	R	R	R	R	R	R	R	R	R	R	R	R	R	R	R	R	S
Ab 38	Blood	R	R	R	R	R	R	R	R	R	R	R	R	R	R	R	R	R	R	S
Ab 39	Tracheal Secretion	R	R	R	R	M	R	R	R	R	R	R	R	R	R	R	R	R	R	S
Ab 40	Tracheal Secretion	R	R	R	R	S	S	R	M	R	R	R	R	R	R	R	S	R	S	S

NOTE: AP – Ampicillin; SXT - Trimethoprim-sulfamethoxazole; SAM - Ampicillin sulbactam; AMC - Amoxicillin clavulanate; GN – Gentamicin; NET – Netilmicin; CXM – Cefuroxime; CAZ – Ceftazidime; CRO – Ceftriaxone; CTX – Cefotaxime; PIP – Piperacillin; CIP – Ciprofloxacin; IPM – Imipenem; MEM – Meropenem; FEP – Cefepime; SCF - Cefoperazone sulbactam; TZP - Piperacillin tazobactam; AN – Amikacin; CT – Colistin; R – resistant; IN/M - intermediate; S – Susceptible. '-' – not available.
